# Prescription of antibacterial agents for acute upper respiratory tract infections in Beijing, 2010–2012

**DOI:** 10.1007/s00228-015-1997-6

**Published:** 2015-12-26

**Authors:** Yiqun Wu, Chao Yang, Hanxu Xi, Yang Zhang, Zijun Zhou, Yonghua Hu

**Affiliations:** Department of Epidemiology and Biostatistics, School of Public Health, Peking University Health Science Centre, 38 Xueyuan Rd., Haidian district, Beijing, 100191 People’s Republic of China; Department of Health Policy and Management, School of Public Health, Peking University Health Science Centre, Beijing, China

**Keywords:** Respiratory tract infections, Antibacterial agents, Prescriptions, Outpatients

## Abstract

**Purpose:**

The purpose of this study was to describe the prescription of antibacterial agents for acute upper respiratory tract infections (URIs) in Beijing.

**Methods:**

A total of 8,588,699 outpatient cases in tertiary hospitals with acute upper respiratory tract infections (URIs) were selected from the Beijing Medical Claim Data for Employees (BMCDE) from Oct 2010 to Sep 2012. Second-generation cephalosporins, third-generation cephalosporins, fourth-generation cephalosporins, fluoroquinolones, macrolides (except for erythromycin), combinations of penicillins (including β-lactamase inhibitors), and streptomycins were classified as broad-spectrum antibacterial agents. The rates for antibiotic prescriptions and broad-spectrum antibiotic use were calculated in all cases as well as in various URI diagnosis subgroups and age (18–44, 45–64, and ≥65 years) subgroups. The most frequently prescribed antibiotic classes were identified by calculating the proportions of the different agents in all prescribed antibiotic agents.

**Results:**

Overall, the rate of antibiotic prescription is 39.0 %, and cases diagnosed with acute tonsillitis, sinusitis, and epiglottitis have the highest prescription rate (73.6 %), followed by acute laryngitis and bronchitis (52.3 %), acute pharyngitis (40.1 %), and acute nasopharyngitis (37.2 %). Broad-spectrum agents were chosen in 82.4 % of the cases that were prescribed antibiotics, ranging from 81.9 % of cases with naspharyngitis to 87.1 % of the cases with tonsillitis, sinusitis, and epiglottitis. Second-generation cephalosporins, macrolides, fluoroquinolones, third-generation cephalosporins, and combinations of penicillins were most frequently prescribed, accounting for more than 80 % of all prescribed antibacterials.

**Conclusions:**

Antibacterial drug prescription for outpatients with acute URIs is common in tertiary hospitals in Beijing, and the prescribed antibacterials are usually broad-spectrum agents.

**Electronic supplementary material:**

The online version of this article (doi:10.1007/s00228-015-1997-6) contains supplementary material, which is available to authorized users.

## Introduction

Acute upper respiratory tract infections (URIs) are the most common infectious illnesses in the general population, and they are the leading cause of missed days at work or school. They represent the most frequent acute diagnosis in the office setting [1]. Acute URIs include the common cold, laryngitis, pharyngitis, tonsillitis, sinusitis, and epiglottitis, and the symptoms commonly include runny nose, cough, sore throat, nasal congestion, headache, low-grade fever, sneezing, and malaise. URIs are largely self-limiting, and the majority of these infections are viral and have no cure [2]. Although the available evidence has shown that antibiotics probably provide little benefit for a large proportion of respiratory tract infections, antibiotics are still largely inappropriately used in clinics [3]. Extensive data have shown that there is excessive antibiotic prescription and overuse of newer broad-spectrum antibiotics for acute URIs in many regions [4–7].

Overprescription of antibiotics is costly, exposes patients to potential side effects and is a major contributor to emerging antibiotic resistance [8–10]. In Europe and the USA, efforts utilizing public health campaigns [11], provider education, and practice guidelines [12] have attempted to reduce antibiotic use with varying degrees of success [7, 13, 14]. In China, increasing attention in recent years has been focused on the misuse of antibiotics, and regulations for proper antibiotic use have been established. However, a basic profile for antibiotic use is limited. In this study, we will describe the prescription of antibacterial agents for outpatients with acute URIs in tertiary hospitals in Beijing.

## Methods

### Data source

Beijing Medical Claim Data for Employees (BMCDE) were used, which contain medical claim data for all working or retired employees who are covered by basic medical insurance in Beijing. Anonymized information on the patient demographic characteristics (age and sex), clinical diagnosis, medications, and reimbursement information were included. Clinical diagnoses were presented in the forms of the International Classification of Disease edition 10 (ICD-10) as well as descriptive texts. Details on the dispensed medications consisted of the branded and generic drug names, formulations, fees, and dispensing date. Ethical approval is not required for the use of encrypted retrospective information.

### Study population

Outpatient cases who were diagnosed with acute URIs in tertiary hospitals were included in the analysis.

The inclusion criteria were the following: (1) outpatient visits in tertiary hospitals; (2) age ≥ 18 years; (3) diagnoses of acute nasopharyngitis (common cold) (ICD-10J00), acute sinusitis (ICD-10J01), acute pharyngitis (ICD-10 J02), acute tonsillitis (ICD-10J03), acute laryngitis and tracheitis (ICD-10J04), acute obstructive laryngitis and epiglottitis (ICD-10J05), acute upper respiratory infections of multiple and unspecified sites (ICD-10J06); (4) complete medication records; and (5) visits between October 1, 2010 and September 30, 2012.

The exclusion criteria were the following: (1) doubtful cases of acute URIs, such as “fever of unknown origin” or “acute tonsillitis to be confirmed” and (2) follow-up visits within 1 month of a prior visit.

### Drug classification

Antibacterial agents were sorted according to the Anatomical Therapeutic Chemical (ATC) classification system (WHO, version 2015 [15]). Second-generation cephalosporins (J01DC), third-generation cephalosporins (J01DD), fourth-generation cephalosporins (J01DE), fluoroquinolones (J01MA), macrolides (J01FA, except for erythromycin J01FA01), combinations of penicillins (including β-lactamase inhibitors (J01CR)), and streptomycins (J01GA) were classified as broad-spectrum antibacterial agents. All other antibiotics were classified as narrow-spectrum, mainly including erythromycin (J01FA01), penicillins with extended spectrum (J01CA), first-generation cephalosporins (J01DB), and other antibacterials.

### Analysis

The baseline demographic characteristics are shown as the means (and standard deviations) for continuous variables and as the numbers (and percentages) for categorical measures. The rates for antibiotic prescriptions were calculated as cases prescribed with one or more antibacterial agents divided by all cases. The broad-spectrum antibiotic use proportions were calculated as the percentage of cases prescribed with broad-spectrum antibacterial agents divided by the cases prescribed with all types of antibacterial agents. All of the above results were calculated in all cases as well as in the various URI diagnosis subgroups and age (18–44, 45–64, and ≥65 years) subgroups. Cases with acute tonsillitis, sinusitis, and epiglottitis were combined in one subgroup due to the small sample sizes. The most frequently prescribed antibiotic classes were identified by calculating the proportions of the different agents of all antibiotic agents that were used. All of the analyses were performed using the Statistical Analysis System software, version 8.2 (SAS Institute, Cary, NC, USA).

## Results

### Basic characteristics

8,588,699 cases with acute URIs were selected in this study. The mean age was 57.6 ± 14.7 years; 19.0 % were 18 to 44 years old, 49.3 % were 45 to 64 years old, and 31.7 % were 65 years and above. Male cases accounted for 39.4 % of total cases. Most of the cases (81.9 %) were diagnosed with acute nasopharyngitis (common cold or acute upper respiratory infections of multiple and unspecified sites). Details of the age, sex distribution, and disease classification of the cases are shown in Table 1.

**Table 1:** Basic characteristics of the cases with acute URIs

	18–44 years		45–64 years		65–years		Total	
	*N*	%	*N*	%	*N*	%	*N*	%
Gender								
Male	594,487	36.4	1,439,734	34.0	1,353,244	49.8	3,387,465	39.4
Female	1,036,899	63.6	2,797,899	66.0	1,366,436	50.2	5,201,234	60.6
URIs subgroups								
Acute nasopharyngitis	1,276,584	78.3	3,493,597	82.4	2,262,610	83.2	7,032,791	81.9
Acute pharyngitis	226,329	13.9	449,971	10.6	241,207	8.9	917,507	10.7
Acute laryngitis and bronchitis	75,303	4.6	241,358	5.7	193,780	7.1	510,441	5.9
Acute tonsillitis, sinusitis, and epiglottitis	53,170	3.3	52,707	1.3	22,083	0.8	127,960	1.6

A more detailed description of the study population is given in the supplementary materials.

### Antibiotic prescription rate

**Fig. 1:**
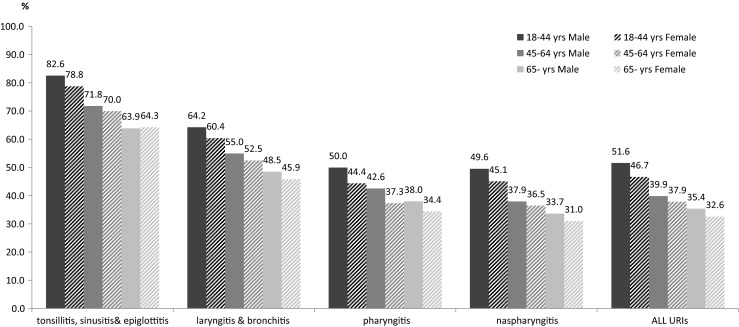
Antibiotic prescription rates for cases with acute URIs

Of all cases, 3,347,423 cases received antibacterial agents with an antibiotic prescription rate of 39.0 %. Cases with acute tonsillitis, sinusitis, and epiglottitis had the highest antibiotic prescription rates (73.6 %), which was followed by acute laryngitis and bronchitis (52.3 %), acute pharyngitis (40.1 %), and acute nasopharyngitis (37.2 %). The rate of antibiotic prescriptions was 34.0 % for the cases aged 65 years and older, and this was lower than rates in the cases who were 45 to 64 years of age (38.5 %) and 18 to 44 years of age (48.5 %). For all URI diagnosis subgroups, the rates of antibiotic prescriptions in the 18–44 years of age subgroup were all higher than rates in the older groups, as seen in Fig. 1. The rates of antibiotic prescriptions in the male and female cases were 73.2 % and 69.7 %, respectively. More men received antibiotic treatment than women (Fig. 1).

### Broad-spectrum antibiotic use

Broad-spectrum antibiotics were commonly prescribed, but they varied across age and diagnosis subgroups. Broad-spectrum agents were chosen in 82.4 % of the cases that were prescribed antibiotics, ranging from 81.9 % of the cases with nasopharyngitis to 87.1 % of cases with tonsillitis, sinusitis, and epiglottitis. For all diagnostic subgroups, the proportion of broad-spectrum antibiotics used in cases 18–44 years of age was the highest, while the proportion in cases older than 64 years of age was the lowest. The proportion of broad-spectrum antibiotic use in men was higher than in women (Table 2).

**Table 2:** Proportion of broad-spectrum antibiotic use in the cases using antibiotics

Gender	Diagnosis	18–44 years	45–64 years	65 years	Total
Male					
	Acute tonsillitis, sinusitis, and epiglottitis	88.4	86.5	86.2	87.3
	Acute laryngitis and bronchitis	88.9	87.0	86.0	86.9
	Acute nasopharyngitis	85.5	82.5	81.1	82.7
	Acute pharyngitis	83.8	82.7	81.3	82.6
	All URIs	85.6	83.0	81.7	83.1
Female					
	Acute tonsillitis, sinusitis, and epiglottitis	88.2	86.2	84.9	87.0
	Acute laryngitis and bronchitis	88.3	86.2	85.1	86.3
	Acute nasopharyngitis	84.5	81.1	79.0	81.4
	Acute pharyngitis	83.3	81.4	79.9	81.7
	All URIs	84.7	81.6	79.8	82.0

### Categories of antibacterial agents

For cases in which antibacterial agents were prescribed, 91.0 % received a single antibacterial agent, whereas the remainder of cases received two or more kinds of antibacterial agents. Among all categories of antibiotics, second-generation cephalosporins (J01DC) are the most commonly used, accounting for 32.8 %. These are followed by macrolides (J01FA, accounting for 18.7 %), fluoroquinolones (J01MA, accounting for 15.4 %), third-generation cephalosporins (J01DD, accounting for 9.0 %), and combinations of penicillins (J01CR, accounting for 6.4 %), as illustrated in Table 3.

**Table 3:** Categories of antibacterial agents used for cases with acute URIs

ATC 4th level, chemical subgroup	ATC 5th level, chemical substance	Percent^a^
J01DC second-generation Cephalosporins	J01DC02 cefuroxime	20.3
	J01DC04 cefaclor	9.0
	Others	3.5
	J01DC total	32.8
J01FA macrolides	J01FA10 azithromycin	8.5
	J01FA01 erythromycin	4.6
	Others	5.6
	J01FA total	18.7
J01MA fluoroquinolones	J01MA12 levofloxacin	11.4
	Others	4.0
	J01MA total	15.4
J01DD third-generation cephalosporins	J01DD15 cefdinir	4.6
	J01DD08 cefixime	3.8
	Others	0.6
	J01DD Total	9.0
J01CR combinations of penicillins, including beta-lactamase inhibitors	J01CR02 amoxicillin and enzyme inhibitor	6.3
	Others	0.1
	J01CR total	6.4
Others		17.7

^a^Percentages of specific agents in all antibiotic agents that are used

With respect to second-generation cephalosporins, the most commonly used antibiotics were cefuroxime and cefaclor. With respect to third-generation cephalosporins, the most commonly used antibiotics were cefdinir and cefixime. The most commonly used fluoroquinolone was levofloxacin. The most commonly used macrolides were azithromycin and erythromycin (Table 3).

## Discussion

Although substantial research suggests that antibiotics probably provide little benefit for a large proportion of respiratory tract infections, the results of this study show that nearly 40 % of outpatients with acute URIs received antibacterial drug prescriptions in tertiary hospitals in Beijing. Antibacterial drug prescriptions are common in tertiary hospitals in Beijing for cases with acute URIs, which is similar to results in the USA [13, 16] and some European countries [17]. In 2011, the European Surveillance of Antimicrobial Consumption project (ESAC) published a set of relevant evidence-based disease-specific quality indicators for outpatient antibiotic use. The acceptable range for the percentage of adult patients with acute URIs who were prescribed antibacterials is 0–20 % [18]. The total prescription rates of antibiotics in this study were much higher than the threshold recommended by the ESAC. The study results also show that different types of URIs have different antibacterial prescription rates. Cases of acute tonsillitis, sinusitis, and epiglottitis had the highest rate of antibacterial prescription (73.6 %), whereas cases with acute nasopharyngitis had the lowest rate (37.2 %). In these data, even the lowest antibiotic prescription rate for acute nasopharyngitis is almost two times the ESAC threshold. In this study, there were different antibiotic prescription rates across age and sex subgroups. Females and older cases with URIs had lower rates than males and young adult cases. These results are similar to other studies [13, 16].

The current study results implicate that antibacterials for URIs in tertiary hospitals in Beijing are usually broad-spectrum, new generation agents. According to the ESAC quality indicators, extended spectrum penicillins and β-lactamase-sensitive penicillins should account for 80–100 % antibiotic prescriptions, and quinolones for no more than 5 % [18]. In this study, penicillins with extended spectrum and β-lactamase-sensitive penicillins accounted for only about 3.4 %, while fluoroquinolones accounted for approximately 15 %, which is threefold higher than the recommendation. Broad-spectrum agents accounting for about 80 % of all prescribed antibacterials. The constitution of antibacterials in this study is largely different from the ESAC recommendations, and it is also different from the antibacterials that are used in the USA and Europe. In the United States, doxycycline, macrolides, and fluoroquinolones have a prominent position as first-line agents for the outpatient treatment of respiratory tract infections, whereas patients in Europe generally receive β-lactam agents [17]. The differences between the antibacterials used in different regions may be related to the different clinically relevant infections, patients’ and physician’s choices, commercial pressures, and differences in regulation. Cephalosporins are not the first-line drugs for respiratory infections, while in this study, approximately 40 % of the cases received second- and third-generation cephalosporins. This is similar to other studies, wherein the use of newer broad-spectrum cephalosporins in the USA and many European countries has increased to a level of inappropriate use [17, 19].

There are limitations in this study. First, only the primary diagnosis was examined in these data. When we performed the analysis, antibiotic prescriptions were attributed to the first diagnosis, while antibiotics might also have been prescribed for the second or third diagnosis, especially for those with other infectious comorbidities. This might have resulted in misclassification, potentially over-representing the frequency of antibiotic prescription for URIs. Second, because our data did not capture the dose for each prescription, we were unable to assess antibiotic consumption in quantity, which could otherwise provide more precise information on the prescribing of antibiotic agents. Third, the patient population in this sample is not representative of all acute URIs, limiting the generalizability. Patients in the BMCDE data only include working or retired employees and residents who are covered by medical insurance; unemployed residents with no medical insurance are not represented in this population. When using these results, caution should be exercised.

Although there are limitations, to the best of our knowledge, this is the first study that uses administrative data to describe the use of antibacterial agents for acute URIs in Beijing. According to these results, antibacterial drug prescriptions for outpatients with acute URIs are common in tertiary hospitals in Beijing, and the most commonly prescribed antibacterials are mostly broad-spectrum agents. Because antimicrobial resistance is a major global public health problem and antibiotic consumption is increasingly recognized as the main reason for resistance [8, 9], judicious antibiotic prescription is critical for preserving antibiotic effectiveness. Public health officials, providers, and policymakers should lead the efforts to promote appropriate antibiotic use. Many European Union countries impose policy restrictions or perform national awareness campaigns to decrease inappropriate antibiotic prescription [17, 20]. Antibacterial consumption has decreased in some of these countries [7]. Reductions in the US antibiotic prescription rates have been observed since the launch of the Centers for Disease Control and Prevention’s “Get Smart: Know When Antibiotics Work” campaign [14]. These successful experiences should be adapted to improve the antibiotic use in China. Further studies on antibacterial prescriptions in primary health care settings, among emergency patients and inpatients, antibiotic consumption doses, and factors associated with antibiotic consumptions in China are needed.

## Electronic supplementary material

ESM 1 (DOCX 15 kb)
