# Supplementary material for: Prescription of antibacterial agents for acute upper respiratory tract infections in Beijing, 2010–2012
Source: Eur J Clin Pharmacol. 2015 Dec 26;72:359–64. doi: 10.1007/s00228-015-1997-6 (PMC4751214; doi:10.1007/s00228-015-1997-6)
Supplement: Supplementary file 1 — (DOCX 15 kb) [file 228_2015_1997_MOESM1_ESM.docx]

Supplementary materials:

In the study period, the database contains medical claim data for about 12.8 million working or retired employees covered by basic medical insurance in Beijing. There were 2,616,340 persons had URIs diagnosis in the study period, and about 65.9% (1,724,401) of them received antibiotics once or more, adding up to 3,347,423 antibiotic prescriptions in total. Patients distribution according to the number of antibiotic prescriptions were seen as below (table S1).

**Table S1 Patients distribution according to the number of antibiotic prescriptions**

| number of  antibiotic prescriptions | patients | |
| --- | --- | --- |
|  | N | % |
| 0 | 891939 | 34.1 |
| 1 | 919880 | 35.2 |
| 2 | 394164 | 15.1 |
| 3 | 200518 | 7.7 |
| ≥4 | 209839 | 8.0 |
| Total | 2616340 | 100.0 |
